# Identification and expression pattern analysis of the *OsCBL* gene family in rice

**DOI:** 10.3389/fpls.2025.1625014

**Published:** 2025-07-09

**Authors:** Zhao Hu, Fengpu Xie, Lu Cai, Run Qian

**Affiliations:** ^1^ College of Biological Sciences and Technology, Taiyuan Normal University, Taiyuan, China; ^2^ Shaanxi University of Chinese Medicine, Shaanxi, China; ^3^ School of Traditional Chinese Materia Medica, Shenyang Pharmaceutical University, Shenyang, China

**Keywords:** rice (*Oryza sativa L*.), OsCBL, genome-wide, abiotic stress, expression patterns

## Abstract

Calcium (Ca^2+^) signaling, which is mediated by calcineurin B-like proteins (CBLs), plays a pivotal role in the way that plants respond to abiotic stresses. However, the systematic bioinformatics and expression pattern analysis of *OsCBLs* remain largely unknown. In this study, we conducted a thorough genome-wide analysis of the *OsCBL* gene family in rice, identifying ten members spanning six chromosomes. Phylogenetic analysis classified these genes into three subfamilies, revealing evolutionary conservation with *Zea mays* and *Hordeum vulgare*, while also highlighting lineage-specific expansions. Structural characterization revealed that all OsCBL proteins contain three conserved EF-hand domains, though variations in protein stability, subcellular localization and motif composition were observed, particularly with regard to the chloroplast localization of OsCBL4/8/9/10. Promoter analysis revealed distinct *cis*-elements linked to responses to abscisic acid, drought and light, suggesting functional diversification among the different members. Expression profiling revealed that *OsCBL5* and *OsCBL9* were specifically induced by heat but repressed by cold. In contrast, *OsCBL3* responded to various stresses, including salt and temperature. Predictions of protein-protein interactions linked several OsCBLs to ion transporters. Notably, the functions of OsCBL1 and OsCBL9 may be exerted through the formation of a heterodimer. These findings provide critical insights into the functional divergence of *OsCBL* genes and lay the groundwork for future studies on calcium-mediated stress responses in rice.

## 1 Introduction

Rice (*Oryza sativa L.*) is the primary food source for over half of the world’s population, making its productivity essential for global food security (Zheng and Qian, 2024). However, rice production faces significant challenges from various abiotic stresses, including drought, salinity and extreme temperatures. These stresses collectively account for over 50% of annual yield losses (Ray et al., 2019). In the context of climate change, these stresses are projected to become more frequent and intense, posing an even greater threat to rice cultivation (Lesk et al., 2016). Therefore, understanding the molecular mechanisms underlying rice stress responses is essential for developing stress-resilient varieties through molecular breeding or genetic engineering approaches.

At the cellular level, plants perceive environmental stress and transmit these signals through complex signaling networks. Calcium ions (Ca²^+^) are ubiquitous secondary messengers in these pathways (Kim et al., 2024). The calcineurin B-like (CBL) protein family, which consists of calcium sensors, plays a pivotal role in decoding Ca²^+^ signatures. These proteins specifically interact with CBL-interacting protein kinases (CIPKs) to regulate downstream responses by phosphorylation pathway (Tang et al., 2020). The CBL-CIPK network has emerged as a central regulatory system modulating various physiological processes, such as ion homeostasis, osmotic adjustment, and stress-responsive gene expression (Yu et al., 2014). In *Arabidopsis thaliana*, AtCBL1 and AtCBL9 regulate drought responses via the ABA signaling pathway (You et al., 2023), and AtCBL4 (SOS3) and AtCIPK24 (SOS2) form a module that mediates salt tolerance (Steinhorst et al., 2022; Gámez-Arjona et al., 2024). In rice, OsCBL1 has been shown to contribute to nitrogen tolerance by regulating *OsNRT2.2* expression (Hu et al., 2023), and OsCBL8 has been found to play a role in the response to drought stress (Gao et al., 2022). It is worth mentioning that over-expression of *OsCBL3/2* in *Arabidopsis thaliana* increases salt tolerance. Despite the findings of these studies, the precise mechanisms through which part of the CBL functions in response to abiotic stresses in rice remain largely unknown. Therefore, a comprehensive analysis and understanding of the potential functions of CBL in abiotic stress is of utmost importance.

Actually, although there is some literature analyzing *OsCBL* genes information (Gu et al., 2008; Kanwar et al., 2014), but these analyses are not comprehensive and in-depth enough. In this study, we performed a comprehensive genome-wide analysis of the *OsCBL* gene family in rice, yielding numerous new findings and insights. This analysis included an examination of phylogenetic relationships, gene structures, conserved motifs, and chromosomal distributions. Then, we systematically investigated the expression patterns of all *OsCBL* genes under various abiotic stresses, including heat, cold, drought and salt treatments, using RNA-seq data. Our results revealed that different *OsCBL* members exhibit distinct stress-responsive expression patterns. For example, *OsCBL5* and *OsCBL9* are specifically induced by heat stress but repressed by cold stress, suggesting their potential roles in temperature perception. In contrast, *OsCBL3* displayed broad responsiveness to multiple stresses, suggesting its involvement in general stress response pathways. These findings offer a new perspective on the *OsCBL* gene and its potential functions in the abiotic stress response in rice, providing a more comprehensive and in-depth understanding.

## 2 Materials and methods

### 2.1 Chromosome localization and gene structure analysis

The *OsCBL* gene sequences in rice were retrieved from the NCBI database (Enter CBL and *Oryza sativa* into the NCBI website to find their corresponding gene names in the rice genome. Then, use these gene names to find the corresponding *OsCBL* family sequences in the rice genome). The structures of the identified genes were checked using IGV-GSAman (v0.9.51) (Chen et al., 2021). Each gene is designated with the prefix *Os* for *Oryza sativa*, followed by CBL to identify the gene family, and a numerical suffix (e.g., *OsCBL1-OsCBL10*). Chromosomal localization and gene structure analysis were performed using TBtools software (Chen et al., 2023). The physical positions of the *OsCBL* genes were determined for chromosomal mapping based on Rice Genome Annotation Project (RGAP) data, while the gene structures were analyzed by comparing the coding sequences with the corresponding genomic DNA sequences.

### 2.2 Protein sequence alignment, conserved domain and conserved motif analysis

A multiple sequence alignment was performed using Clustal Omega and visualized in Jalview. Conserved domain analysis was performed using the SMART (http://smart.embl-heidelberg.de/) and InterProScan (https://www.ebi.ac.uk/interpro/search/sequence/) websites. MEME-based motif discovery was performed with the following parameters: ten motifs with a width of 6-50 and an E-value of 1e-10 were identified via the MEME website (http://meme-suite.org/).

### 2.3 Phylogenetic relationships and gene duplication analysis

The amino acid (AA) sequences of CBL proteins, including those from *Oryza sativa*, *Zea mays* and *Hordeum vulgare*, were aligned to generate a phylogenetic tree. This was done using the neighbour-joining (NJ) method in MEGA12 software with 1000 replications for bootstrapping. Gene duplication analysis using MCScanX identified both whole-genome and tandem duplication events.

### 2.4 Subcellular localization, protein 3D structure prediction and PPI analysis

Subcellular localization was analysis by WoLF (https://wolfpsort.hgc.jp/) online website. Protein 3D structure prediction for *OsCBL* gene families using the AlphaFold 3 (https://alphafoldserver.com/). We employed the STRING database (https://cn.string-db.org/) to predict potential interaction partners of OsCBL proteins. Subsequently, we utilized Cytoscape to visualize and analyze the resulting network.

### 2.5 Promoter analysis

The 2.0 kb promoter sequences located in the upstream region of each *OsCBL* gene were retrieved from the Rice Genome database using the TBtools software. A systematic analysis of these promoter regions was conducted using the PlantCARE database (Lescot et al., 2002) to identify putative cis-regulatory elements.

### 2.6 Expression analysis

Expression data were obtained from the Rice Genome Annotation Project (https://rice.uga.edu/index.shtml). RNA-seq datasets comprising rice samples subjected to stress treatment (e.g. drought, salt, heat and cold) with three biological replicates per condition were retrieved from NCBI. Raw data concerning tissue expression can be obtained from the NCBI (https://www.ncbi.nlm.nih.gov/) at the following accession number: PRJNA482217. Raw data concerning heat stress can be obtained from NCBI (PRJNA604026). Raw data concerning cold stress can be obtained from the NCBI under the accession number PRJNA827493. Raw data concerning salt stress can be obtained from NCBI under the accession number PRJNA1037192. According to the NCBI, raw data concerning drought stress can be obtained from PRJNA306542. The RNA-seq data were processed according to (Hu et al., 2024). In brief, the raw reads were quality-trimmed using Trimmomatic and aligned to the NIP reference genome (ftp://ftp.ensemblgenomes.org/pub/plants/release-44/fasta/oryza_sativa/dna/) using STAR software. Differential expression analysis was performed using DESeq2, applying thresholds of |log2FC| > 1 and padj < 0.05.

### 2.7 Data analysis

Experimental data were collected to calculate the mean and standard error of the mean (SEM) for three biological replicates. Statistical significance between different samples was determined using a Student’s *t*-test at *p* < 0.05. All statistical analyses were performed using Prism 9 software.

## 3 Results

### 3.1 Chromosomal localization and physiochemical properties of *OsCBL* genes

**Figure 1:**
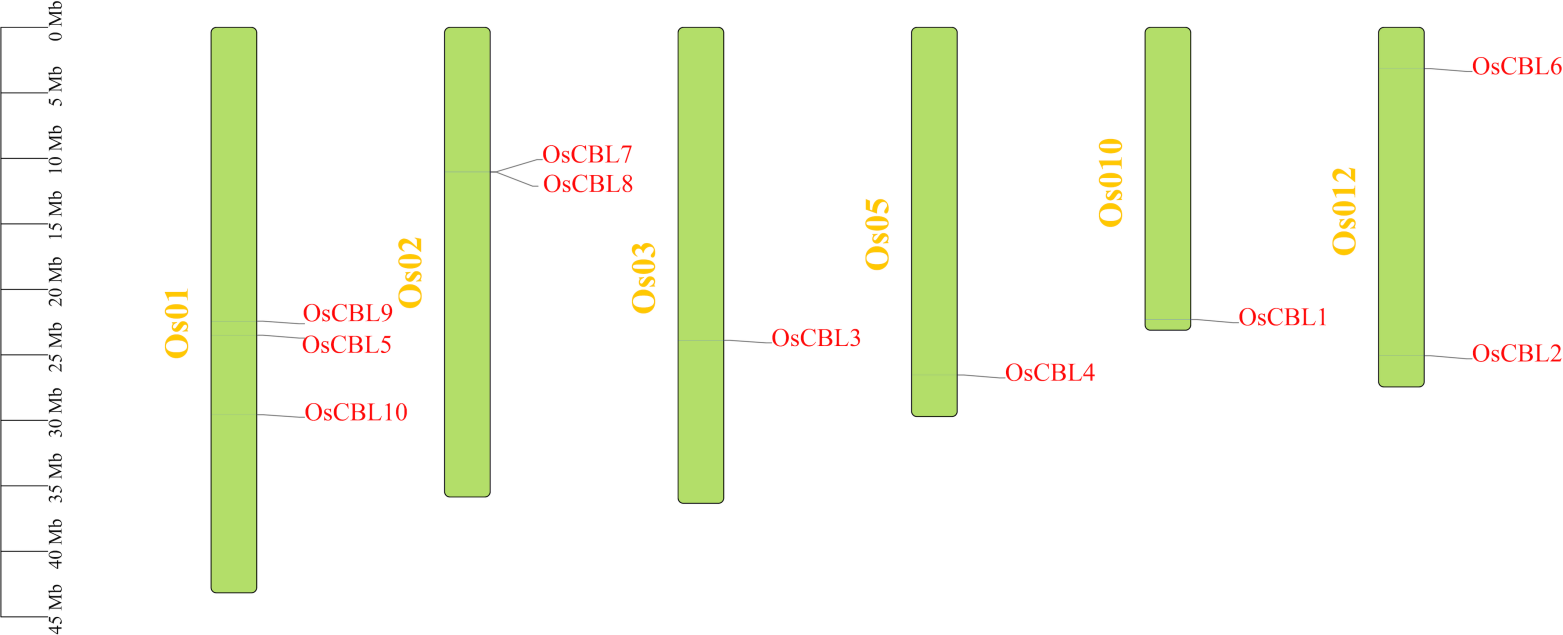
Distribution of *OsCBL* genes in rice chromosomes. Only show the chromosomes where located the *OsCBL* gene. The yellow font represents chromosome numbers and Green arc length indicate chromosome size.

A total of ten *CBL* genes (**Supplementary Table 1**) have been identified in rice, encoding proteins ranging from 210 to 290 amino acids in length and from 23916.07 to 32892.48 Daltons in molecular weight (**Table 1**). The shortest protein is OsCBL4, while the longest is OsCBL9. The isoelectric point and hydrophobicity of OsCBL ranged from 4.66 to 4.98 and -0.336 to -0.058, respectively (**Table 1**). The negative values of hydrophobicity imply that all OsCBL proteins are hydrophilic. The instability index, a quantitative metric for evaluating protein stability, employs a threshold of 40 for hydrophobicity to categorize proteins as stable or unstable, respectively. A total of six proteins were identified as stable, including OsCBL1, OsCBL2, OsCBL3, OsCBL5, OsCBL7 and OsCBL8. The remaining four proteins, namely OsCBL4, OsCBL6, OsCBL9 and OsCBL10 were deemed unstable (**Table 1**). Chromosomal localization analysis revealed the distribution of the 10 *OsCBL* genes across six chromosomes. Specifically, *OsCBL5*, *OsCBL9*, and *OsCBL10* were localized to chromosome 1, *OsCBL7* and *OsCBL8* to chromosome 2, *OsCBL2* and *OsCBL6* to chromosome 12, and *OsCBL3*, *OsCBL4*, and *OsCBL1* to chromosomes 3, 5 and 10, respectively (**Figure 1**; **Supplementary Table 1**).

**Table 1:** Protein information of OsCBL family.

Gene name	Number of amino acid	Molecular weight (Da)	Theoretical pI	Instability index	Aliphatic index	Grand average of hydropathicity
*OsCBL1*	213	24496.85	4.66	35.46	86.01	-0.259
*OsCBL2*	225	25864.5	4.8	35.7	94.84	-0.216
*OsCBL3*	225	25803.52	4.77	39	95.29	-0.228
*OsCBL4*	210	23916.07	4.98	58.61	79.48	-0.336
*OsCBL5*	218	25088.46	4.79	37.25	87.61	-0.28
*OsCBL6*	226	25879.4	4.86	42.39	88.89	-0.323
*OsCBL7*	213	24399.79	4.78	32.1	93.47	-0.224
*OsCBL8*	213	24490.98	4.92	37.77	91.6	-0.204
*OsCBL9*	290	32892.48	4.84	42.23	95.24	-0.058
*OsCBL10*	266	29915.14	4.87	44.97	96.47	-0.111

### 3.2 Gene structure and protein structure analysis of *OsCBL*

**Figure 2:**
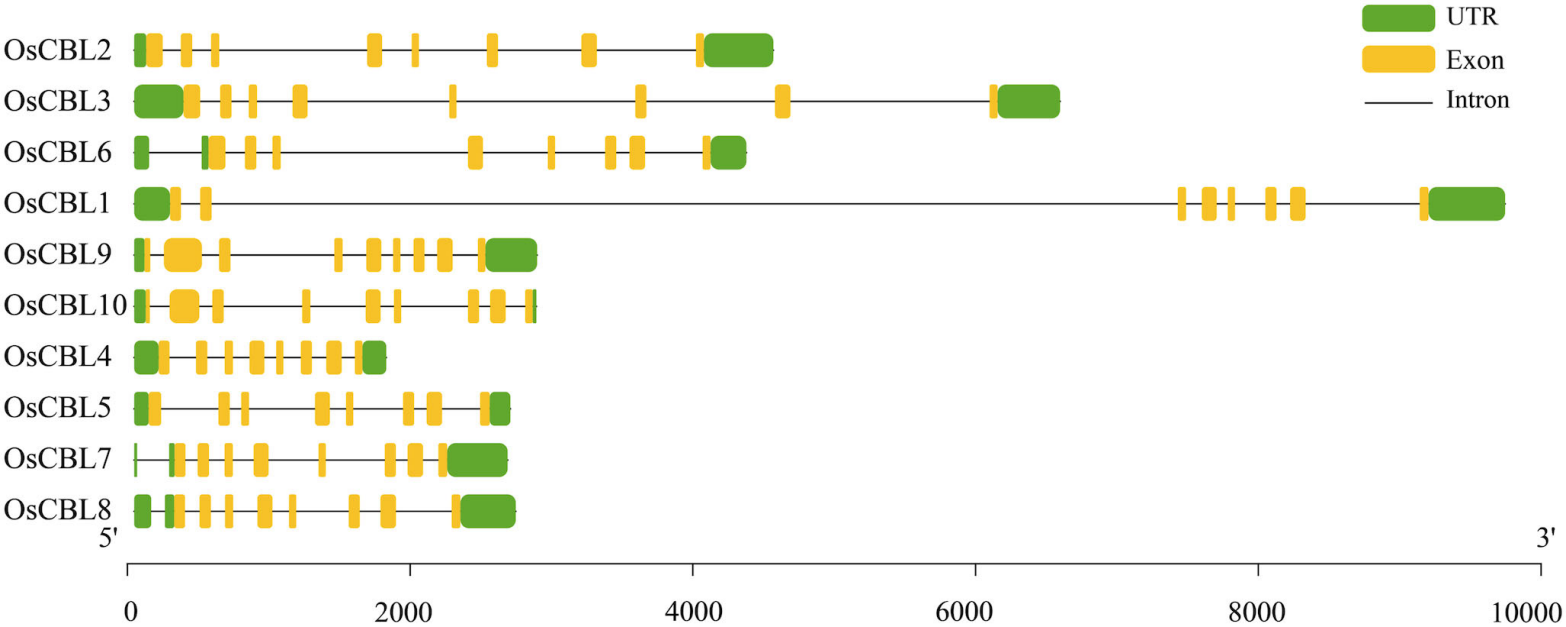
Gene structure analysis of the *OsCBL*. Green boxes indicate UTR, yellow boxes indicate exons and black lines indicate introns.

**Figure 3:**
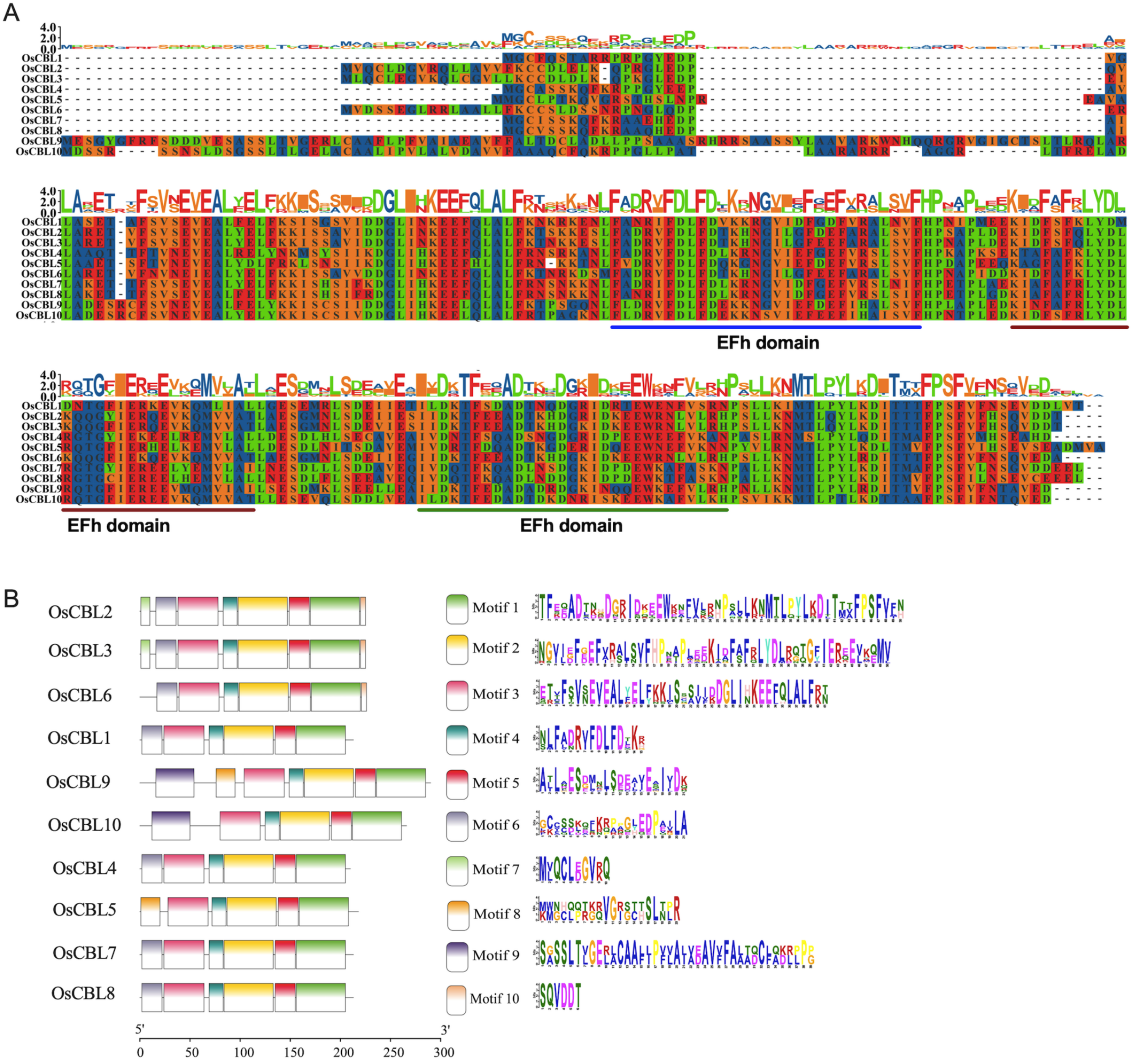
Protein sequence alignment, conserved domain and conserved motif analysis of the OsCBL. **(A)** Protein sequence alignment and conserved domain analysis of the OsCBL. **(B)** Conserved motif analysis of the OsCBL. Different colours indicate different motifs.

The gene structures of *OsCBL* exhibit substantial conservation, characteristically comprising eight or nine exons (**Figure 2**). *OsCBL9* and *OsCBL10* were found to contain nine exons, while the remaining *OsCBL* genes exhibited an exon count of eight (**Figure 2**). Multiple sequence alignment and conserved domain analysis of OsCBL proteins revealed a high degree of amino acid sequence conservation among CBL family members (**Figure 3A**). In addition, all OsCBL proteins contained three Ca²^+^-binding domain (EFh domain) (**Figure 3A**), indicating evolutionary conservation of functional domains among OsCBL proteins. In order to provide further elucidation regarding the evolution of OsCBL members, MEME analysis identified ten conserved motifs (motif 1~motif 10) (**Figure 3B**). The analysis revealed that all OsCBL members contained motif 1~motif 5. In addition, all OsCBL proteins contain motif 6, with the exception of OsCBL5, OsCBL9 and OsCBL10. Furthermore, the analysis revealed that OsCBL2 and OsCBL3 contain motif 7, OsCBL9 and CBL5 contain motif 8, OsCBL9 and OsCBL10 contain motif 9, and OsCBL2, OsCBL3 and OsCBL6 contain motif 10 (**Figure 3B**).

### 3.3 Phylogenetic relationships, gene duplication and synteny analysis of *OsCBL*

**Figure 4:**
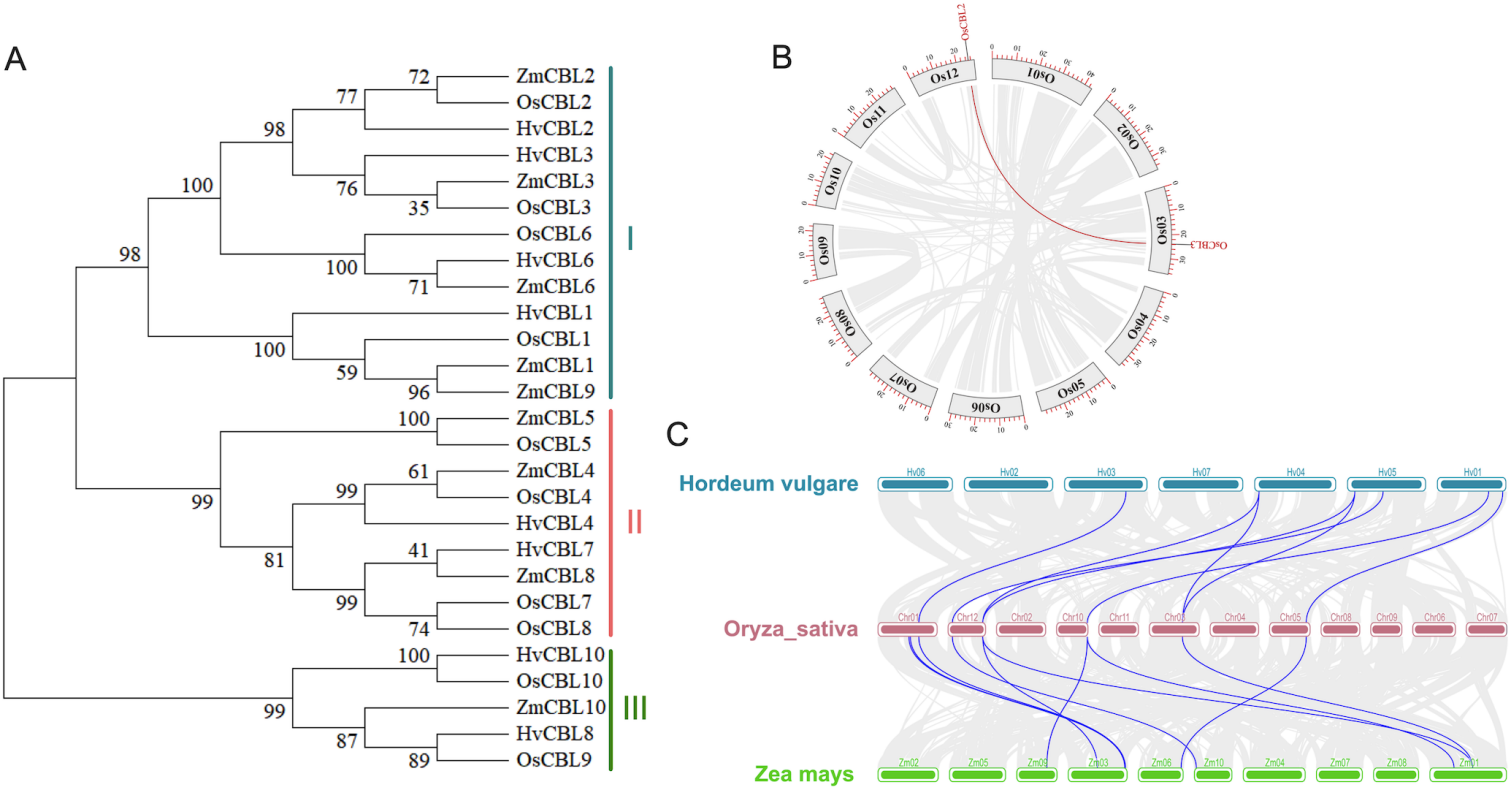
Phylogenetic relationships and gene duplication analysis of OsCBL. **(A)** Phylogenetic relationships analysis of CBL in *Oryza sativa*, *Zea mays* and *Hordeum vulgare*. **(B)** Gene segmental duplication analysis of *OsCBL* in rice. Gray lines indicate all synteny blocks in the rice genome, and the red lines indicate duplicated *OsCBL* gene pairs. The chromosome number is indicated at the gray arc. The scale at the periphery of the chromosome represents the physical location (Kb). **(C)** Synteny analysis of *CBL* genes in the genomes between *Oryza sativa* and *Hordeum vulgare* or *Zea mays*. The gray lines show collinear blocks. The blue lines indicate the syntenic gene pairs between *Oryza sativa* and *Hordeum vulgare* or *Zea mays*, respectively.

To elucidate the evolutionary characteristics of OsCBL proteins, we performed a comprehensive phylogenetic analysis using amino acid sequences from rice OsCBL members, along with key CBL homologs from *Zea mays* and *Hordeum vulgare*. The results demonstrated that the OsCBL proteins could be classified into three subfamilies according to their affinities (**Figure 4A**). The OsCBL2, OsCBL3, OsCBL6 and OsCBL1 proteins were classified into subfamily I, while the OsCBL4, OsCBL5, OsCBL7 and OsCBL8 proteins were classified into subfamily II, and the OsCBL9 and OsCBL10 proteins were classified into subfamily III. The OsCBL family members demonstrate a high degree of similarity to the CBL family members of *Zea mays* and *Hordeum vulgare*, suggesting the potential for conserved physiological and biochemical functions. Furthermore, synteny relationship analysis of OsCBL genes revealed collinearity among two out of the ten OsCBLs (**Figure 4B**). To further elucidate their evolutionary relationships, a syntenic maps were constructed between *Oryza sativa* and *Zea mays*, *Hordeum vulgare*. A total of eight homologous CBL gene pairs were identified in the comparison of *Oryza sativa* with both *Zea mays*, *Hordeum vulgare* (**Figure 4C**). These results suggest that *Oryza sativa* is closely related to *Zea mays* and *Hordeum vulgare*.

### 3.4 Subcellular localization and 3D structure prediction of OsCBL

**Figure 5:**
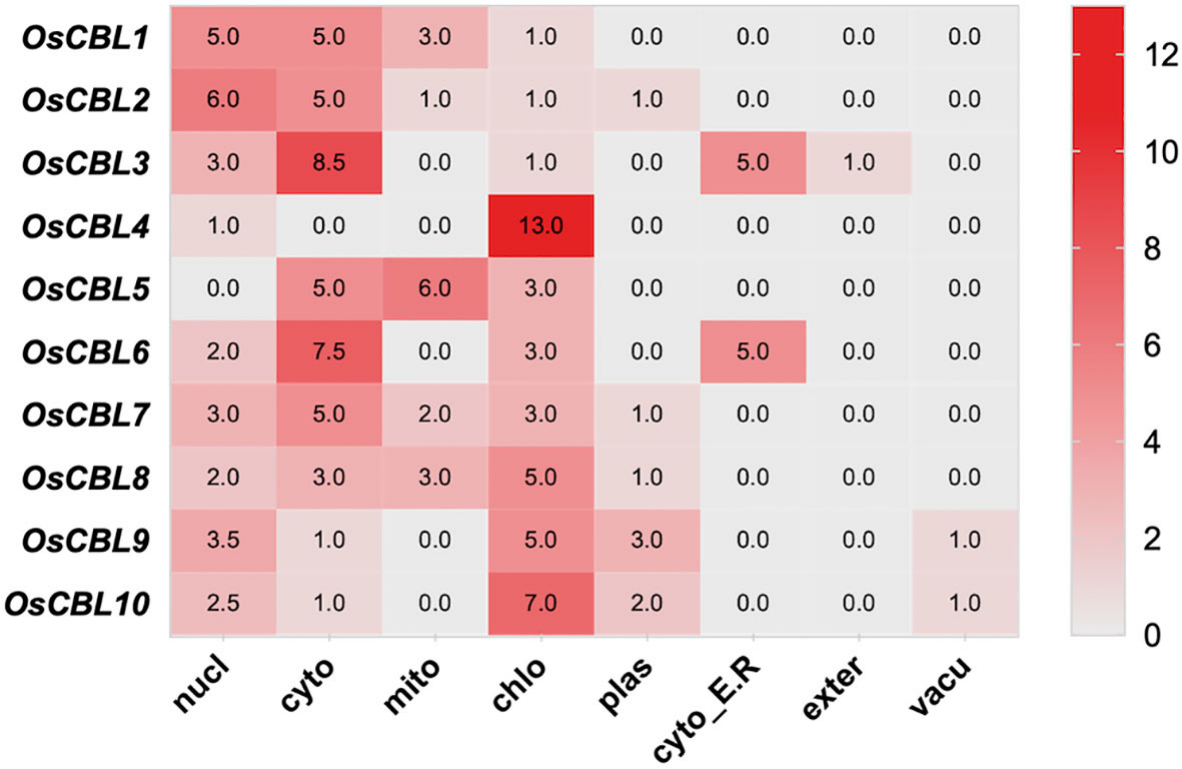
The subcellular localization prediction analysis of OsCBL proteins. The number indicates the prediction score.

The subcellular localization prediction analysis was conducted to determine the distribution of OsCBL proteins within cells (**Figure 5**). OsCBL1 and OsCBL2 were predominantly localized to the cytoplasm and nucleus, whereas OsCBL3 and OsCBL6 exhibited dual localization to the cytoplasm and cytoplasmic membrane. Interestingly, OsCBL5 was localized to the cytoplasm and mitochondria, whereas OsCBL7 was localized to the cytoplasm. In contrast, the remaining members (OsCBL4, OsCBL8, OsCBL9 and OsCBL10) were predicted to localize to chloroplasts, suggesting potential roles in plastid-related signaling or stress responses.

**Figure 6:**
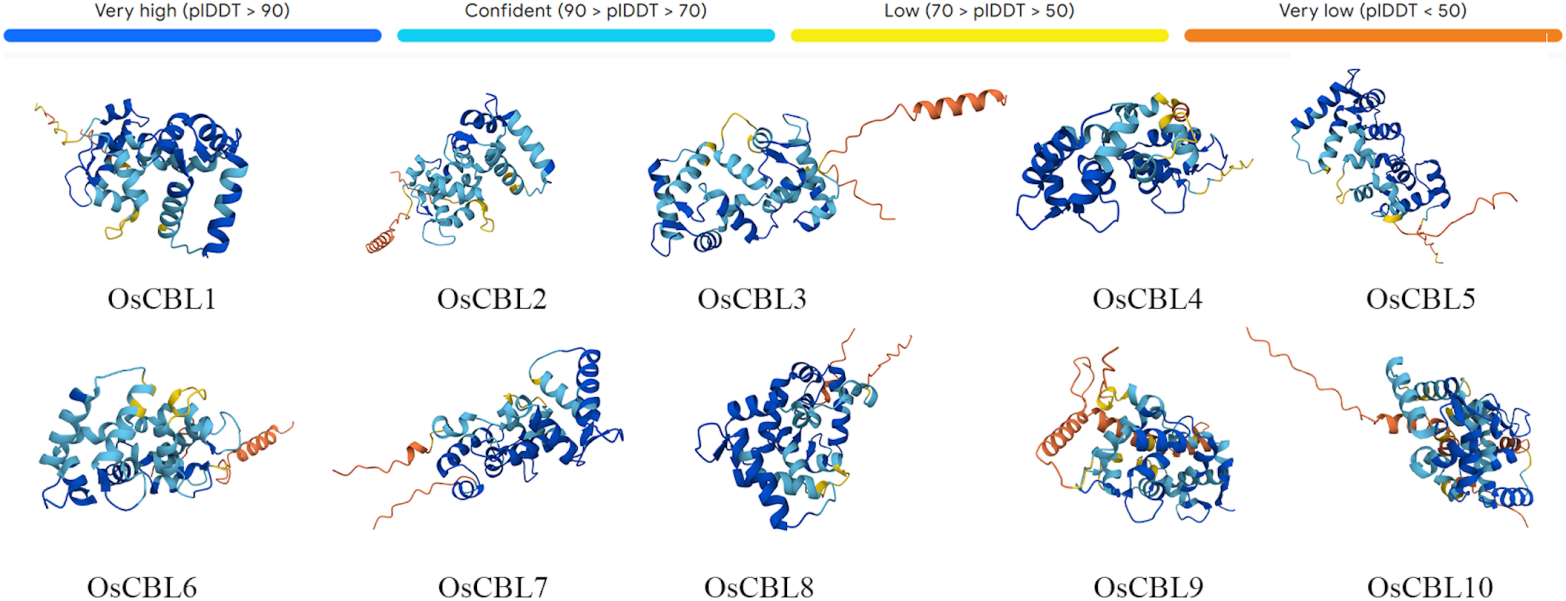
Protein 3D structure prediction model of OsCBL proteins. AlphaFold produces a per-residue confidence score (pLDDT) between 0 and 100.

The predicted 3D structures of all OsCBL proteins exhibit a conserved architectural framework, characterized by the canonical EF-hand domain (**Figure 6**). Each domain comprises two calcium-binding loops arranged in a helix-loop-helix motif, a hallmark feature of calcium sensor proteins. Structural alignments revealed high similarity across OsCBL members, suggesting evolutionary conservation of the calcium-binding machinery.

### 3.5 Protein-protein interaction analysis

**Figure 7:**
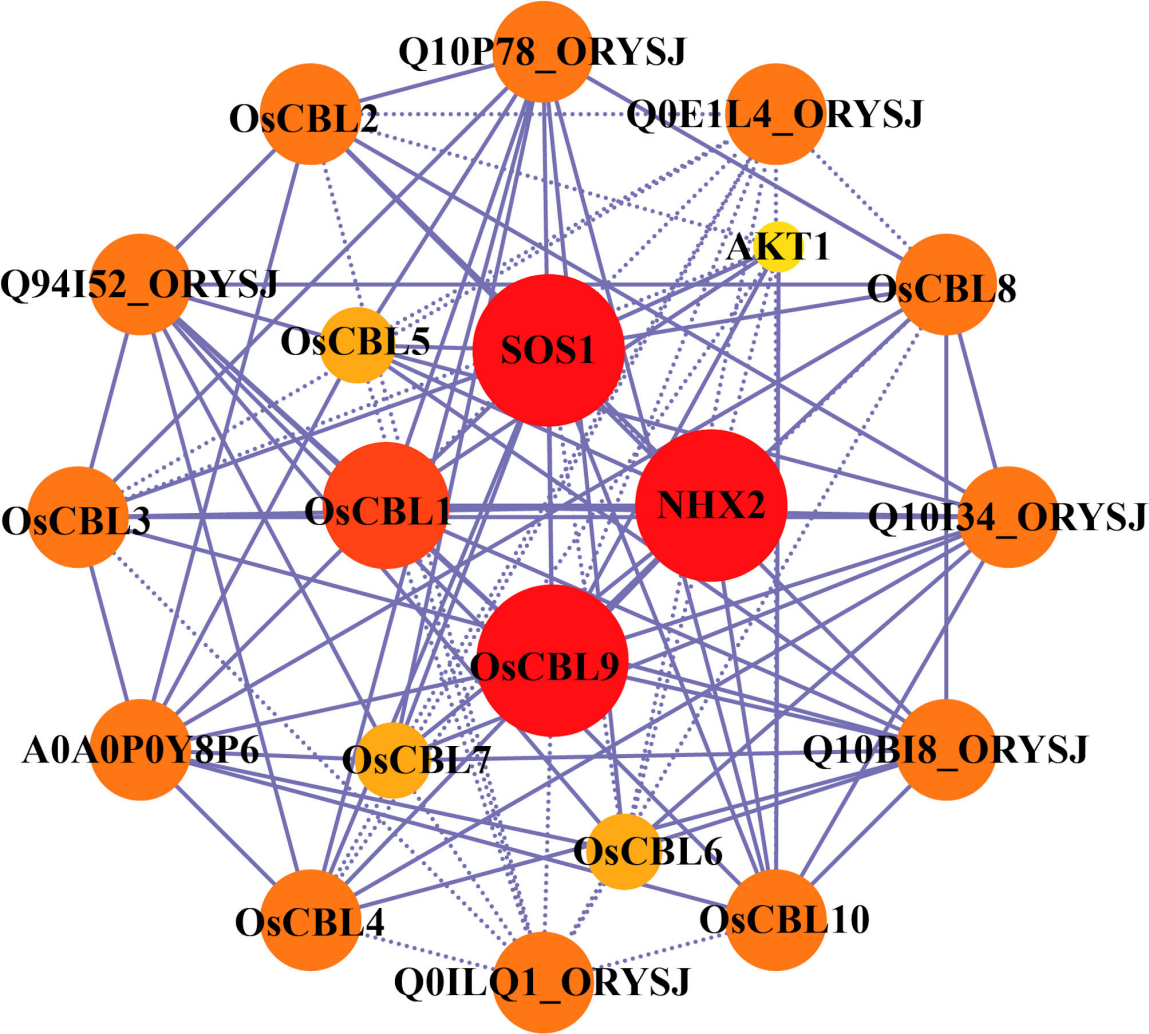
Protein-protein interaction network assembly of OsCBL proteins. The area and color of the circles represent the number of interacting proteins, the larger area and darker color of the circles indicate more interacting proteins. The dashed lines represent protein interaction score ≤ 0.5, and solid lines means protein interaction score > 0.5.

In order to systematically investigate the functional roles of CBL proteins in rice, the protein-protein interaction network of OsCBLs was predicted and analyzed using the STRING database (**Figure 7**; **Supplementary Table 2**). The results demonstrate that several OsCBLs interact with key ion transporters, thereby implicating their roles in abiotic stress adaptation. For instance, OsCBL10 and OsCBL4 showed interactions with OsSOS1 (scores 0.782 and 0.667, respectively), a critical component of the salt overly sensitive (SOS) pathway, while OsCBL1/2/3/9 bound to the potassium channel OsAKT1 (scores 0.459–0.788), indicating cross-talk between calcium and potassium homeostasis. Furthermore, interactions with OsNHX2 (e.g., OsCBL9-OsNHX2, score 0.646) suggested potential roles in ion compartmentalization. Intriguingly, based predictions revealed OsCBL proteins can interact with each other, such as OsCBL1-OsCBL9 (score 0.845) and OsCBL8-OsCBL9 (score 0.437), suggesting that they may form heterodimers to regulate signaling. However, some lower-confidence interactions (e.g., OsNHX2-OsSOS1, score 0.42) and associations with uncharacterized proteins (e.g., Q0E1L4, Q10P78) require further experimental validation.

### 3.6 Prediction of *cis*-elements in *OsCBL* genes promoters

**Figure 8:**
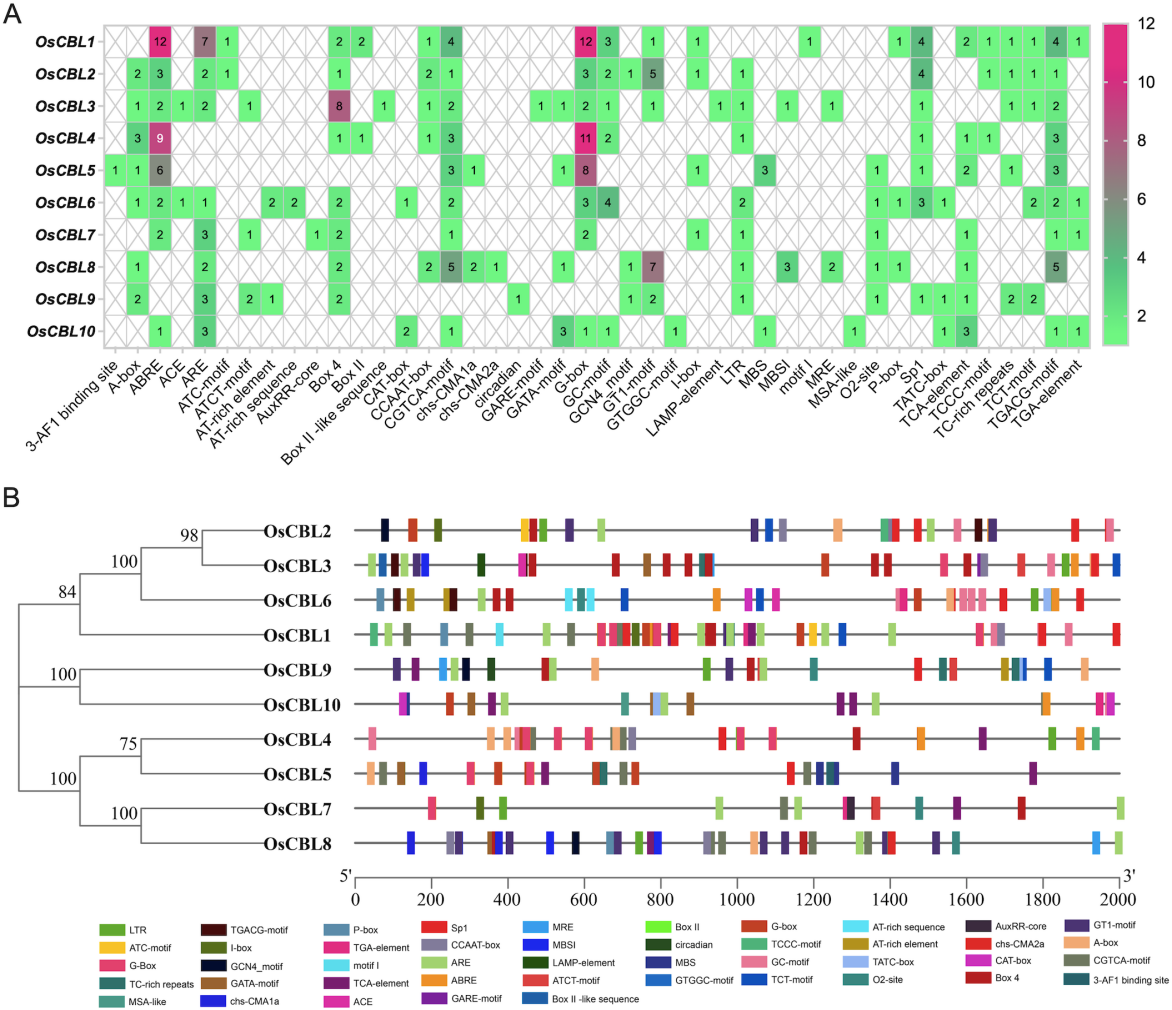
Predicted *cis*-elements in *OsCBL* promoter. **(A)** The number of *cis*-elements in each *OsCBL* gene promoter. **(B)** The distribution of these *cis*-elements across the *OsCBL* promoters. Promoter sequences (-2Kb) of 10 *OsCBL* were analyzed by PlantCARE. Different *cis*-elements are represented by different colors.

To explore the underlying function of the *OsCBL* genes, we performed a comprehensive analysis of *cis*-elements in the 2 kb promoter regions upstream of the *OsCBL* genes in rice (**Figure 8**). The *OsCBL1*, *OsCBL4* and *OsCBL5* promoters showed a particularly high number of *cis*-elements, indicating a strong responsiveness to abscisic acid signaling and drought stress. Several stress-related elements were identified, including anaerobic response elements (ARE) in *OsCBL1*, *OsCBL7*, *OsCBL9* and *OsCBL10*. The analysis also revealed significant light regulation potential through abundant G-box elements in *OsCBL1*, *OsCBL4* and *OsCBL5* promoters and Box 4 elements in *OsCBL3* promoter, as well as GT1 motif in *OsCBL2* and *OsCBL8* promoters and Sp1 in *OsCBL1* and *OsCBL2* promoters. In addition, a high number of MeJA-related elements (CGTCA motif and TGACG motif) are found in *OsCBL1* and *OsCBL8* promoters. These distinct *cis*-elements profiles suggest functional specialization among *OsCBL* family members, with *OsCBL1*, *OsCBL4* and *OsCBL5* possibly focused on ABA and light responses. The results provide a valuable basis for future experimental studies of *OsCBL* gene regulation and function in rice.

### 3.7 Expression patterns of *OsCBL* genes in different rice tissues

**Figure 9:**
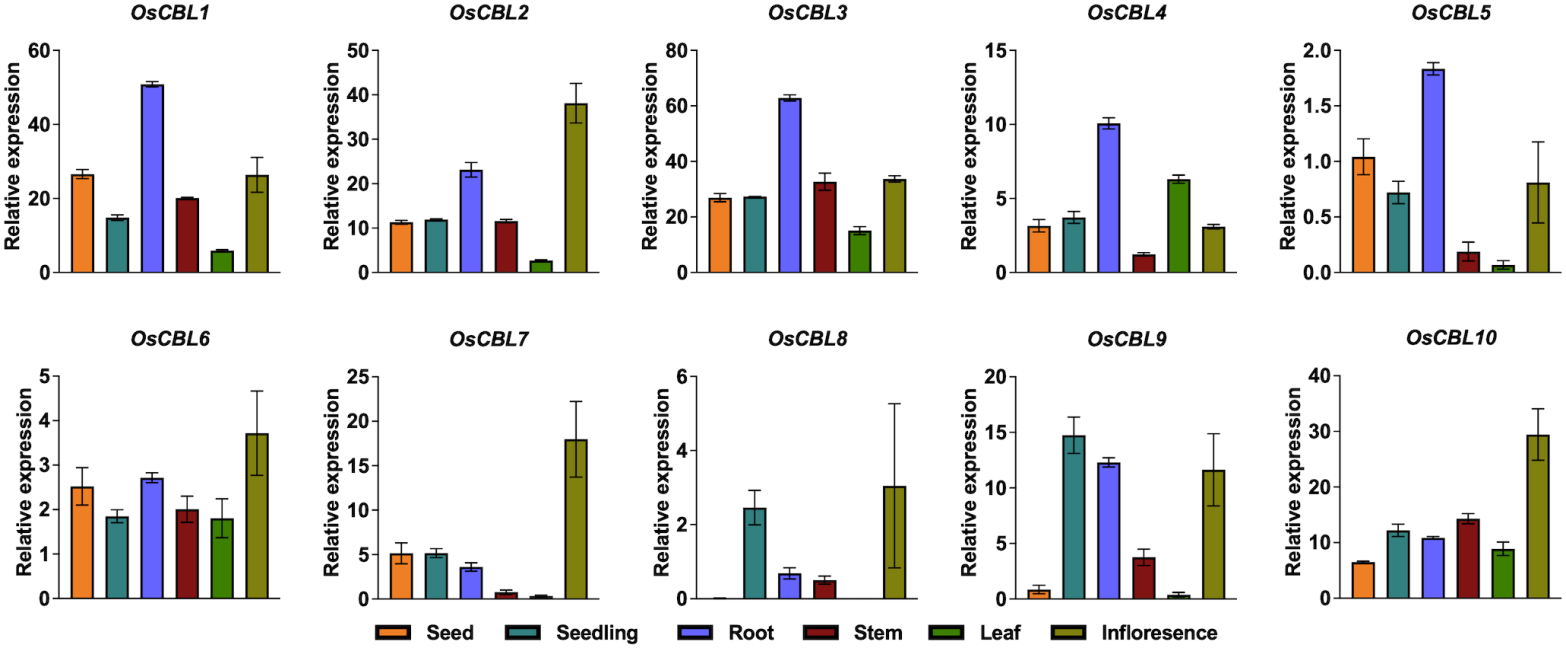
Expression analysis of the *OsCBL* in different tissues. The expression data were obtained from the Rice Genome Annotation Project (https://rice.uga.edu/index.shtml).

Based on the RNA-seq analysis, it demonstrated that *OsCBL1*, *OsCBL2*, *OsCBL3*, *OsCBL4* and *OsCBL5* were expressed predominantly in the root system (**Figure 9**), suggesting the possibility of a key role for these genes in root development and stress response. Aboveground tissues demonstrated that *OsCBL2, OsCBL6*, *OsCBL7*, *OsCBL8*, *OsCBL9* and *OsCBL10* was expressed at a higher level in infloresence (**Figure 9**), suggesting a potential role in reproductive development. Furthermore, the expression value of the *OsCBL3*, *OsCBL6* and *OsCBL10* was found to be greater than five in all tissues, whereas other *OsCBL* did not demonstrate this result, suggesting the possibility that *OsCBL3*, *OsCBL6* and *OsCBL10* may play a critical function in different tissues.

### 3.8 Gene expression profiling under various stress conditions

**Figure 10:**
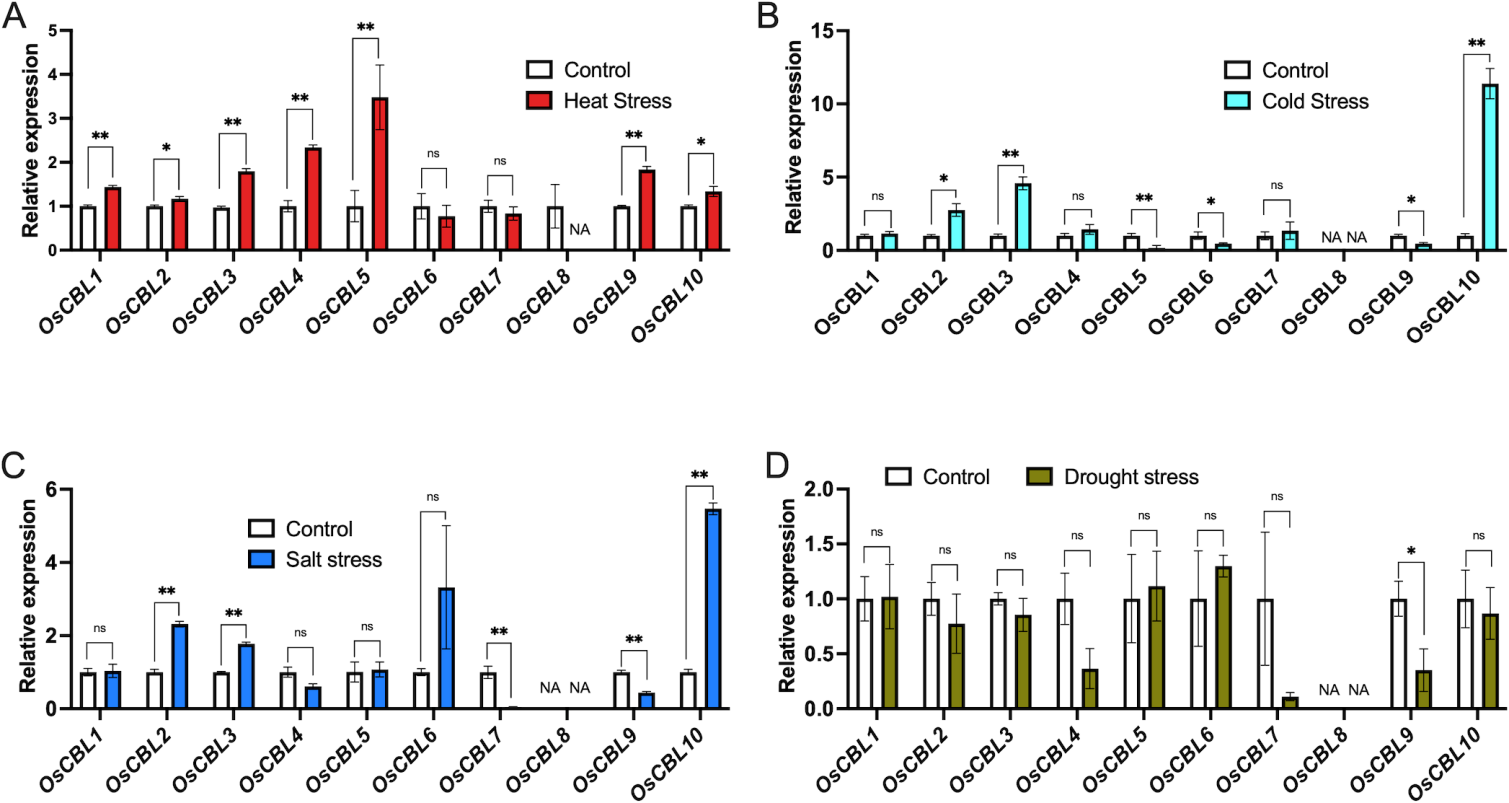
Expression analysis of the OsCBL under Heat **(A)**, Cold **(B)**, Salt **(C)**, Drought **(D)** stress conditions. The control was defined as “1”. The expression data were obtained from the Rice Genome Annotation Project (https://rice.uga.edu/index.shtml). NA indicates “not available”. n =3 biologically independent samples. The error bars represent ± SEM. *p < 0.05, **p < 0.01 compared to control (Student’s *t*-test). “ns” indicates no significant difference.

Given the abundance of abiotic stress-associated *cis*-elements in the promoter region of *OsCBL* genes, and to further investigate the potential role of *OsCBL* genes in abiotic stress response, we analyzed their expression patterns under drought, salinity, high and low temperature stress conditions using RNA-seq data. The results demonstrated that the stress response expression profiles of *OsCBL* family members exhibited variation (**Figure 10**). The induction of heat stress resulted in the expression of all *OsCBLs* with the exception of *OsCBL6*, *OsCBL7* and *OsCBL8* (**Figure 10A**). In the context of cold stress treatment, the expression of *OsCBL2*, *OsCBL3* and *OsCBL10* was found to be enhanced, while the expression of *OsCBL5*, *OsCBL6* and *OsCBL9* was repressed (**Figure 10B**). It is noteworthy that *OsCBL5* and *OsCBL9* exhibited contrasting regulatory patterns under diverse stress conditions, with heat inducing its activity while cold repressing it. Furthermore, salt stress induced the expression of *OsCBL2*, *OsCBL3* and *OsCBL10*, while salt stress repressed the expression of *OsCBL7* and *OsCBL9* (**Figure 10C**). In addition, under drought stress, only *OsCBL9* expression weas found to be considerably diminished (**Figure 10D**).

## 4 Discussion

Our comprehensive analysis of the *OsCBL* gene family in rice reveals conserved features and lineage-specific adaptations in this essential calcium signaling component. Identifying ten *OsCBL* genes distributed across six chromosomes (**Figure 1**; **Supplementary Table 1**) and confirming the presence of conserved EF-hand domains (**Figure 3A**) establishes their evolutionary relationship with CBL families in other plants (Kudla et al., 1999). However, distinct subfamily classification (**Figure 4A**) and variable motif compositions (**Figure 3B**) suggest functional diversification, particularly for subfamily III members (OsCBL9/10), which exhibit unique structural features, such as extended amino acid sequences and chloroplast localization (**Figure 3**, **Figure 5**). However, the localization of CBL family proteins is not focused on a particular location in the cell. This spatial and functional specialization suggests that different CBL families evolved to interpret spatially restricted calcium signals in response to specific environmental stimuli (Kudla et al., 2018; Plasencia et al., 2020). In addition, our computational predictions, which are based on amino acid sequences, suggest that OsCBL proteins are relatively unlikely to be localized at the plasma membrane. This finding contrasts with the experimentally verified localization of some plasma membrane-localized CBLs in other species. This is may be because prediction tools cannot adequately account for dynamic post-translational modifications (e.g., acetylation), protein-protein interactions (e.g., with CIPKs), stress conditions or timing, all of which are known to mediate subcellular localization (Plasencia et al., 2020; Guo et al., 2023).

The predicted protein-protein interaction network (**Figure 7**) reveals both conserved and novel potential partnerships. Interactions between OsCBL4 and OsSOS1 components suggest the maintenance of salt tolerance mechanisms similar to those in *Arabidopsis* (Gámez-Arjona et al., 2024; Xie et al., 2024). In contrast, novel associations with OsAKT1 indicate an expanded role in potassium that is unique to rice (Li et al., 2014). Furthermore, identifying high-confidence CBL-CBL interactions, such as OsCBL1-OsCBL9 in rice (**Figure 7**), reveals a fascinating layer of complexity in calcium signaling networks.

Promoter analysis (**Figure 8**) revealed striking differences in the composition of *cis*-elements that correlate with the observed expression patterns (**Figure 10**). The enrichment of *OsCBL1/4/5* in ABA-responsive elements aligns with their stress induction. Tissue-specific expression (**Figure 9**) further supports functional divergence, with root-predominant *OsCBL1/2/3/4/5* potentially regulating ion uptake, and reproductive tissue-enriched *OsCBL2/6/7/8/9/10* influencing developmental processes. The differential expression patterns observed under abiotic stress (**Figure 10**) reveal striking functional specialization among the *OsCBL* members. The present study provides evidence that members of the *OsCBL* gene family play crucial, yet distinct, roles in the way rice responds to abiotic stresses, particularly in response to temperature fluctuations. Our findings demonstrate that *OsCBL5* and *OsCBL9* are specifically induced by heat stress but repressed by cold stress (**Figure 10**), which suggests that they have a specialized function in temperature perception. The temperature-specific induction of *OsCBL5* and *OsCBL9* is particularly relevant in the context of climate change, given that rising global temperatures are predicted to double the frequency of heat stress in major rice-growing regions by 2050 (Intergovernmental Panel on Climate C 2023). From an applied perspective, the *OsCBL5* and *OsCBL9* genes could be used to select heat-tolerant varieties or as targets for precision genome editing. Furthermore, the salt-inducible *OsCBL2/3/6/10* and the drought-responsive *OsCBL9* genes can serve as specific molecular targets for crop improvement. These genes’ function may be enhanced through gene editing (e.g., CRISPR-Cas9) or overexpression, approaches that have been successfully applied in other stress-responsive genes. For instance, knockout *OsSPL10* can improve rice’s drought tolerance (Li et al., 2023), whereas overexpressing *OsHMGB707* can increase rice’s drought tolerance (Xu et al., 2021). Additionally, stress-responsive synthetic promoters can be developed using the identified *cis*-elements, a strategy that has been effectively employed to fine-tune gene expression (Mazumder and McMillen, 2014; Tan et al., 2023; Claeys et al., 2024). However, the *cis-*elements on the *OsCBL* promoter were identified only through bioinformatics analysis in this study, and their role in regulating *OsCBL* expression awaits experimental validation. Therefore, further experimental validation of the application of synthetic promoter strategies to the *OsCBL* gene family is required. In addition, our work is limited by the fact that information about the potential function of OsCBL in stress response is based only on systematic bioinformatics analysis. Currently, we are validating these applications through field trials of selected *OsCBL* transgenic materials under realistic stress conditions, thus bridging the gap between molecular discovery and agricultural implementation.

## 5 Conclusion

Our study provides a comprehensive characterization of the genomic and expression patterns of the *OsCBL* gene family in rice, revealing that they may play critical roles in stress adaptation. Ten *OsCBL* genes were identified, all of which exhibit conserved EF-hand calcium-binding domains, but have distinct physicochemical properties, gene structures and subcellular localization patterns. These findings emphasize the functional diversification of OsCBLs in stress adaptation and developmental regulation, and could be valuable for the genetic improvement of stress-tolerant rice varieties.

## Data Availability

The datasets presented in this study can be found in online repositories. The names of the repository/repositories and accession number(s) can be found at: BioProject ID: PRJNA48221, PRJNA604026, PRJNA827493, PRJNA1037192 and PRJNA306542.
