# Supplementary material for: Identification and expression pattern analysis of the OsCBL gene family in rice
Source: Front Plant Sci. 2025 Jul 9;16:1625014. doi: 10.3389/fpls.2025.1625014 (PMC12283654; doi:10.3389/fpls.2025.1625014)
Supplement: Supplementary file 1 [file DataSheet1.pdf]

Supplementary Material

Title: Identification and expression pattern analysis of the *OsCBL* gene family in rice

Zhao Hu[1*], Fengpu Xie[3], Lu Cai [1], Run Qian[2*]

[1] College of Biological Sciences and Technology, Taiyuan Normal University, Taiyuan, China
[2] School of Traditional Chinese Materia Medica, Shenyang Pharmaceutical University, Shenyang, China
[3] Shaanxi University of Chinese Medicine, Shaanxi, China

*** Correspondence:**
Zhao Hu
zhaohu@tynu.edu.cn
Run Qian
qianrunSYPHU@outlook.com

Supplementary Tables

Supplementary Table 1: *OsCBL* family gene information

Gene Name	Gene ID	Accession number	NCBI_Locus
OsCBL1	Os10g0564800	LOC_Os10g41510	LOC4349412
OsCBL2	Os12g0597000	LOC_Os12g40510	LOC4352701
OsCBL3	Os03g0626800	LOC_Os03g42840	LOC4333502
OsCBL4	Os05g0534400	LOC_Os05g45810	LOC4339432
OsCBL5	Os01g0598200	LOC_Os01g41510	LOC4325962
OsCBL6	Os12g0162400	LOC_Os12g06510	LOC4351575
OsCBL7	Os02g0291000	LOC_Os02g18880	LOC107276277
OsCBL8	Os02g0291400	LOC_Os02g18930	LOC107276173
OsCBL9	Os01g0579600	LOC_Os01g39770	LOC9269084
OsCBL10	Os01g0711500	LOC_Os01g51420	LOC4325933

Supplementary Table 2: Protein-protein interaction analysis

node1	node2	combined_score
A0A0P0Y8P6	CBL2	0.543
A0A0P0Y8P6	CBL10	0.543
A0A0P0Y8P6	CBL6	0.543
A0A0P0Y8P6	CBL5	0.543
A0A0P0Y8P6	CBL8	0.543
A0A0P0Y8P6	CBL4	0.543
A0A0P0Y8P6	CBL9	0.543
A0A0P0Y8P6	CBL3	0.543
A0A0P0Y8P6	CBL7	0.543
A0A0P0Y8P6	CBL1	0.543
AKT1	CBL4	0.425
AKT1	NHX2	0.429
AKT1	CBL3	0.459
AKT1	CBL2	0.493
AKT1	CBL10	0.552
AKT1	SOS1	0.567
AKT1	CBL1	0.736
AKT1	CBL9	0.788
CBL1	Q0E1L4_ORYSJ	0.43
CBL1	Q0ILQ1_ORYSJ	0.43
CBL1	Q10BI8_ORYSJ	0.543
CBL1	Q10I34_ORYSJ	0.543
CBL1	Q10P78_ORYSJ	0.577
CBL1	CBL9	0.845
CBL1	SOS1	0.722
CBL1	NHX2	0.651
CBL1	Q94I52_ORYSJ	0.543
CBL10	Q0E1L4_ORYSJ	0.43
CBL10	Q0ILQ1_ORYSJ	0.43
CBL10	Q10BI8_ORYSJ	0.543
CBL10	Q10I34_ORYSJ	0.543
CBL10	Q10P78_ORYSJ	0.575
CBL10	Q94I52_ORYSJ	0.543
CBL10	NHX2	0.624
CBL10	SOS1	0.782
CBL2	Q0E1L4_ORYSJ	0.43
CBL2	Q0ILQ1_ORYSJ	0.43
CBL2	Q10BI8_ORYSJ	0.543
CBL2	Q10I34_ORYSJ	0.543
CBL2	Q10P78_ORYSJ	0.575
CBL2	Q94I52_ORYSJ	0.543
CBL2	NHX2	0.598

CBL2	SOS1	0.639
CBL3	Q0E1L4_ORYSJ	0.43
CBL3	Q0ILQ1_ORYSJ	0.43
CBL3	Q10BI8_ORYSJ	0.543
CBL3	Q10I34_ORYSJ	0.543
CBL3	Q10P78_ORYSJ	0.575
CBL3	SOS1	0.61
CBL3	NHX2	0.528
CBL3	Q94I52_ORYSJ	0.543
CBL4	Q0E1L4_ORYSJ	0.43
CBL4	Q0ILQ1_ORYSJ	0.43
CBL4	Q10BI8_ORYSJ	0.543
CBL4	Q10I34_ORYSJ	0.543
CBL4	Q10P78_ORYSJ	0.575
CBL4	SOS1	0.667
CBL4	NHX2	0.609
CBL4	Q94I52_ORYSJ	0.543
CBL5	Q0E1L4_ORYSJ	0.43
CBL5	Q0ILQ1_ORYSJ	0.43
CBL5	Q10BI8_ORYSJ	0.543
CBL5	Q10I34_ORYSJ	0.543
CBL5	Q10P78_ORYSJ	0.575
CBL5	NHX2	0.521
CBL5	Q94I52_ORYSJ	0.543
CBL5	SOS1	0.615
CBL6	Q0E1L4_ORYSJ	0.43
CBL6	Q0ILQ1_ORYSJ	0.43
CBL6	Q10BI8_ORYSJ	0.543
CBL6	Q10I34_ORYSJ	0.543
CBL6	Q10P78_ORYSJ	0.575
CBL6	NHX2	0.487
CBL6	SOS1	0.495
CBL6	Q94I52_ORYSJ	0.543
CBL7	Q0E1L4_ORYSJ	0.43
CBL7	Q0ILQ1_ORYSJ	0.43
CBL7	Q10BI8_ORYSJ	0.543
CBL7	Q10I34_ORYSJ	0.543
CBL7	Q10P78_ORYSJ	0.575
CBL7	Q94I52_ORYSJ	0.543
CBL7	NHX2	0.557
CBL7	SOS1	0.604
CBL8	Q0E1L4_ORYSJ	0.43
CBL8	Q0ILQ1_ORYSJ	0.43
CBL8	Q10BI8_ORYSJ	0.543

CBL8	Q10I34_ORYSJ	0.543
CBL8	Q10P78_ORYSJ	0.575
CBL8	CBL9	0.437
CBL8	NHX2	0.521
CBL8	Q94I52_ORYSJ	0.543
CBL8	SOS1	0.574
CBL9	Q0E1L4_ORYSJ	0.43
CBL9	Q0ILQ1_ORYSJ	0.43
CBL9	Q10BI8_ORYSJ	0.543
CBL9	Q10I34_ORYSJ	0.543
CBL9	Q10P78_ORYSJ	0.575
CBL9	Q94I52_ORYSJ	0.543
CBL9	NHX2	0.646
CBL9	SOS1	0.741
NHX2	SOS1	0.42

Supplementary Table 3: Predicted *cis*-elements in *OsCBL* promoter

Gene	*cis*-element	sequence	position	description
OsCBL9	MRE	AACCTAA	220	MYB binding site involved in light responsiveness
OsCBL9	GCN4_motif	TGAGTCA	280	cis-regulatory element involved in endosperm expression
OsCBL9	Box 4	ATTAAT	488	part of a conserved DNA module involved in light responsiveness
OsCBL9	Box 4	ATTAAT	1025	part of a conserved DNA module involved in light responsiveness
OsCBL9	TCT-motif	TCTTAC	1737	part of a light responsive element
OsCBL9	TCT-motif	TCTTAC	1803	part of a light responsive element
OsCBL9	O2-site	GATGACATGG	1190	cis-acting regulatory element involved in zein metabolism regulation
OsCBL9	GT1-motif	GGTTAAT	99	light responsive element
OsCBL9	GT1-motif	GGTTAA	969	light responsive element
OsCBL9	A-box	CCGTCC	618	cis-acting regulatory element
OsCBL9	A-box	CCGTCC	1899	cis-acting regulatory element
OsCBL9	TCA-element	CCATCTTTTT	148	cis-acting element involved in salicylic acid responsiveness
OsCBL9	circadian	CAAAGATATC	346	cis-acting regulatory element involved in circadian control
OsCBL9	TATC-box	TATCCCA	1727	cis-acting element involved in gibberellin-responsiveness

OsCBL9	ATCT-motif	AATCTAATCC	1053	part of a conserved DNA module involved in light responsiveness
OsCBL9	ATCT-motif	AATCTAATCC	1555	part of a conserved DNA module involved in light responsiveness
OsCBL9	LTR	CCGAAA	910	cis-acting element involved in low-temperature responsiveness
OsCBL9	AT-rich element	ATAGAAATCAA	1690	binding site of AT-rich DNA binding protein (ATBP-1)
OsCBL9	Sp1	GGGCGG	1463	light responsive element
OsCBL9	TC-rich repeats	ATTCTCTAAC	1528	cis-acting element involved in defense and stress responsiveness
OsCBL9	TC-rich repeats	ATTCTCTAAC	1718	cis-acting element involved in defense and stress responsiveness
OsCBL9	ARE	AAACCA	250	cis-acting regulatory element essential for the anaerobic induction
OsCBL9	ARE	AAACCA	507	cis-acting regulatory element essential for the anaerobic induction
OsCBL9	ARE	AAACCA	1058	cis-acting regulatory element essential for the anaerobic induction
OsCBL5	I-box	atGATAAGGTC	111	part of a light responsive element
OsCBL5	MBS	CAACTG	1206	MYB binding site involved in drought-inducibility
OsCBL5	MBS	CAACTG	1246	MYB binding site involved in drought-inducibility
OsCBL5	MBS	CAACTG	1403	MYB binding site involved in drought-inducibility
OsCBL5	GATA-motif	AAGGATAAGG	110	part of a light responsive element
OsCBL5	TGACG-motif	TGACG	63	cis-acting regulatory element involved in the MeJA-responsiveness
OsCBL5	TGACG-motif	TGACG	692	cis-acting regulatory element involved in the MeJA-responsiveness

OsCBL5	TGACG-motif	TGACG	1173	cis-acting regulatory element involved in the MeJA-responsiveness
OsCBL5	chs-CMA1a	TTACTTAA	168	part of a light responsive element
OsCBL5	3-AF1 binding site	TAAGAGAGGAA	1233	light responsive element
OsCBL5	TCA-element	CCATCTTTTT	487	cis-acting element involved in salicylic acid responsiveness
OsCBL5	TCA-element	CCATCTTTTT	1764	cis-acting element involved in salicylic acid responsiveness
OsCBL5	O2-site	GTTGACGTGA	620	cis-acting regulatory element involved in zein metabolism regulation
OsCBL5	ABRE	ACGTG	293	cis-acting element involved in the abscisic acid responsiveness
OsCBL5	ABRE	ACGTG	365	cis-acting element involved in the abscisic acid responsiveness
OsCBL5	ABRE	GACACGTGGC	446	cis-acting element involved in the abscisic acid responsiveness
OsCBL5	ABRE	CACGTG	448	cis-acting element involved in the abscisic acid responsiveness
OsCBL5	ABRE	ACGTG	449	cis-acting element involved in the abscisic acid responsiveness
OsCBL5	ABRE	ACGTG	621	cis-acting element involved in the abscisic acid responsiveness
OsCBL5	A-box	CCGTCC	31	cis-acting regulatory element
OsCBL5	G-box	TACGTG	364	cis-acting regulatory element involved in light responsiveness
OsCBL5	G-box	ACACGTG(G/t)CACC	443	cis-acting regulatory element involved in light responsiveness
OsCBL5	G-box	GCCACGTGGA	446	cis-acting regulatory element involved in light responsiveness
OsCBL5	G-box	CACGTG	448	cis-acting regulatory element involved in light responsiveness
OsCBL5	G-box	CACGTC	621	cis-acting regulatory element involved in light responsiveness
OsCBL5	G-box	CACGAC	723	cis-acting regulatory element involved in light responsiveness
OsCBL5	CGTCA-motif	CGTCA	63	cis-acting regulatory element involved in the MeJA-responsiveness

OsCBL5	CGTCA-motif	CGTCA	692	cis-acting regulatory element involved in the MeJA-responsiveness
OsCBL5	CGTCA-motif	CGTCA	1173	cis-acting regulatory element involved in the MeJA-responsiveness
OsCBL5	Sp1	GGGCGG	1130	light responsive element
OsCBL5	G-Box	CACGTT	292	cis-acting regulatory element involved in light responsiveness
OsCBL5	G-Box	CACGTG	448	cis-acting regulatory element involved in light responsiveness
OsCBL5	TC-rich repeats	GTTTTCTTAC	640	cis-acting element involved in defense and stress responsiveness
OsCBL10	GATA-motif	GATAGGA	294	part of a light responsive element
OsCBL10	GATA-motif	GATAGGA	772	part of a light responsive element
OsCBL10	GATA-motif	GATAGGA	867	part of a light responsive element
OsCBL10	MBS	CAACTG	123	MYB binding site involved in drought-inducibility
OsCBL10	TGA-element	AACGAC	1938	auxin-responsive element
OsCBL10	GTGGC-motif	CATCGTGTGGC	1967	part of a light responsive element
OsCBL10	TGACG-motif	TGACG	1795	cis-acting regulatory element involved in the MeJA-responsiveness
OsCBL10	GC-motif	CCCCCG	1962	enhancer-like element involved in anoxic specific inducibility
OsCBL10	CGTCA-motif	CGTCA	1795	cis-acting regulatory element involved in the MeJA-responsiveness
OsCBL10	G-box	CACGAC	238	cis-acting regulatory element involved in light responsiveness
OsCBL10	TATC-box	TATCCCA	779	cis-acting element involved in gibberellin-responsiveness
OsCBL10	MSA-like	(T/C)C(T/C)AACGG(T/C)(T/C)A	696	cis-acting element involved in cell cycle regulation

OsCBL10	TCA-element	CCATCTTTTT	348	cis-acting element involved in salicylic acid responsiveness
OsCBL10	TCA-element	CCATCTTTTT	1260	cis-acting element involved in salicylic acid responsiveness
OsCBL10	TCA-element	CCATCTTTTT	1293	cis-acting element involved in salicylic acid responsiveness
OsCBL10	CAT-box	GCCACT	115	cis-acting regulatory element related to meristem expression
OsCBL10	CAT-box	GCCACT	1967	cis-acting regulatory element related to meristem expression
OsCBL10	ABRE	GCCGCGTGGC	1799	cis-acting element involved in the abscisic acid responsiveness
OsCBL10	ARE	AAACCA	381	cis-acting regulatory element essential for the anaerobic induction
OsCBL10	ARE	AAACCA	799	cis-acting regulatory element essential for the anaerobic induction
OsCBL10	ARE	AAACCA	1352	cis-acting regulatory element essential for the anaerobic induction
OsCBL7	TGA-element	AACGAC	1276	auxin-responsive element
OsCBL7	I-box	TGATAATGT	317	part of a light responsive element
OsCBL7	Box 4	ATTAAT	1351	part of a conserved DNA module involved in light responsiveness
OsCBL7	Box 4	ATTAAT	1734	part of a conserved DNA module involved in light responsiveness
OsCBL7	TGACG-motif	TGACG	1112	cis-acting regulatory element involved in the MeJA-responsiveness
OsCBL7	AuxRR-core	GGTCCAT	1287	cis-acting regulatory element involved in auxin responsiveness
OsCBL7	CGTCA-motif	CGTCA	1112	cis-acting regulatory element involved in the MeJA-responsiveness
OsCBL7	G-box	CACGTG	191	cis-acting regulatory element involved in light responsiveness
OsCBL7	ABRE	CACGTG	191	cis-acting element involved in the abscisic acid responsiveness
OsCBL7	ABRE	ACGTG	192	cis-acting element involved in the abscisic acid responsiveness

OsCBL7	O2-site	GTTGACGTGA	1466	cis-acting regulatory element involved in zein metabolism regulation
OsCBL7	TCA-element	CCATCTTTTT	1565	cis-acting element involved in salicylic acid responsiveness
OsCBL7	G-Box	CACGTG	191	cis-acting regulatory element involved in light responsiveness
OsCBL7	ARE	AAACCA	943	cis-acting regulatory element essential for the anaerobic induction
OsCBL7	ARE	AAACCA	1149	cis-acting regulatory element essential for the anaerobic induction
OsCBL7	ARE	AAACCA	1993	cis-acting regulatory element essential for the anaerobic induction
OsCBL7	ATCT-motif	AATCTAATCC	1354	part of a conserved DNA module involved in light responsiveness
OsCBL7	LTR	CCGAAA	377	cis-acting element involved in low-temperature responsiveness
OsCBL8	P-box	CCTTTTG	657	gibberellin-responsive element
OsCBL8	GATA-motif	GATAGGA	346	part of a light responsive element
OsCBL8	MRE	AACCTAA	1381	MYB binding site involved in light responsiveness
OsCBL8	MRE	AACCTAA	1929	MYB binding site involved in light responsiveness
OsCBL8	GCN4_motif	TGAGTCA	567	cis-regulatory element involved in endosperm expression
OsCBL8	Box 4	ATTAAT	358	part of a conserved DNA module involved in light responsiveness
OsCBL8	Box 4	ATTAAT	1163	part of a conserved DNA module involved in light responsiveness
OsCBL8	TGACG-motif	TGACG	921	cis-acting regulatory element involved in the MeJA-responsiveness
OsCBL8	TGACG-motif	TGACG	950	cis-acting regulatory element involved in the MeJA-responsiveness
OsCBL8	TGACG-motif	TGACG	1187	cis-acting regulatory element involved in the MeJA-responsiveness

OsCBL8	TGACG-motif	TGACG	1332	cis-acting regulatory element involved in the MeJA-responsiveness
OsCBL8	TGACG-motif	TGACG	1392	cis-acting regulatory element involved in the MeJA-responsiveness
OsCBL8	chs-CMA1a	TTACTTAA	136	part of a light responsive element
OsCBL8	chs-CMA1a	TTACTTAA	366	part of a light responsive element
OsCBL8	TCA-element	CCATCTTTTT	764	cis-acting element involved in salicylic acid responsiveness
OsCBL8	MBSI	aaaAaaC(G/C)GTTA	262	MYB binding site involved in flavonoid biosynthetic genes regulation
OsCBL8	MBSI	TTTTTACGGTTA	500	MYB binding site involved in flavonoid biosynthetic genes regulation
OsCBL8	MBSI	TTTTTACGGTTA	782	MYB binding site involved in flavonoid biosynthetic genes regulation
OsCBL8	GT1-motif	GGTTAA	261	light responsive element
OsCBL8	GT1-motif	GGTTAA	394	light responsive element
OsCBL8	GT1-motif	GGTTAA	677	light responsive element
OsCBL8	GT1-motif	GGTTAA	1059	light responsive element
OsCBL8	GT1-motif	GGTTAA	1115	light responsive element
OsCBL8	GT1-motif	GGTTAA	1379	light responsive element
OsCBL8	GT1-motif	GGTTAAT	1510	light responsive element
OsCBL8	O2-site	GATGA(C/T)(A/G)TG(A/G)	1562	cis-acting regulatory element involved in zein metabolism regulation
OsCBL8	A-box	CCGTCC	1034	cis-acting regulatory element
OsCBL8	CGTCA-motif	CGTCA	921	cis-acting regulatory element involved in the MeJA-responsiveness

OsCBL8	CGTCA-motif	CGTCA	950	cis-acting regulatory element involved in the MeJA-responsiveness
OsCBL8	CGTCA-motif	CGTCA	1187	cis-acting regulatory element involved in the MeJA-responsiveness
OsCBL8	CGTCA-motif	CGTCA	1332	cis-acting regulatory element involved in the MeJA-responsiveness
OsCBL8	CGTCA-motif	CGTCA	1392	cis-acting regulatory element involved in the MeJA-responsiveness
OsCBL8	LTR	CCGAAA	733	cis-acting element involved in low-temperature responsiveness
OsCBL8	chs-CMA2a	TCACTTGA	1394	part of a light responsive element
OsCBL8	CCAAT-box	CAACGG	239	MYBHv1 binding site
OsCBL8	CCAAT-box	CAACGG	911	MYBHv1 binding site
OsCBL8	ARE	AAACCA	1310	cis-acting regulatory element essential for the anaerobic induction
OsCBL8	ARE	AAACCA	1988	cis-acting regulatory element essential for the anaerobic induction
OsCBL3	GATA-motif	GATAGGG	754	part of a light responsive element
OsCBL3	MRE	AACCTAA	920	MYB binding site involved in light responsiveness
OsCBL3	TCT-motif	TCTTAC	1982	part of a light responsive element
OsCBL3	GC-motif	CCCCCG	1811	enhancer-like element involved in anoxic specific inducibility
OsCBL3	MBSI	aaaAaaC(G/C)GTTA	173	MYB binding site involved in flavonoid biosynthetic genes regulation
OsCBL3	LAMP-element	CTTTATCA	320	part of a light responsive element
OsCBL3	A-box	CCGTCC	1922	cis-acting regulatory element
OsCBL3	G-box	CACGAC	1220	cis-acting regulatory element involved in light responsiveness
OsCBL3	CGTCA-motif	CGTCA	94	cis-acting regulatory element involved in the MeJA-responsiveness

OsCBL3	CGTCA-motif	CGTCA	431	cis-acting regulatory element involved in the MeJA-responsiveness
OsCBL3	ATCT-motif	AATCTAATCC	1733	part of a conserved DNA module involved in light responsiveness
OsCBL3	ARE	AAACCA	34	cis-acting regulatory element essential for the anaerobic induction
OsCBL3	ARE	AAACCA	119	cis-acting regulatory element essential for the anaerobic induction
OsCBL3	TC-rich repeats	ATTCTCTAAC	900	cis-acting element involved in defense and stress responsiveness
OsCBL3	Box II -like sequence	TCCGTGTACCA	62	cis-acting regulatory element
OsCBL3	Box 4	ATTAAT	454	part of a conserved DNA module involved in light responsiveness
OsCBL3	Box 4	ATTAAT	672	part of a conserved DNA module involved in light responsiveness
OsCBL3	Box 4	ATTAAT	805	part of a conserved DNA module involved in light responsiveness
OsCBL3	Box 4	ATTAAT	862	part of a conserved DNA module involved in light responsiveness
OsCBL3	Box 4	ATTAAT	915	part of a conserved DNA module involved in light responsiveness
OsCBL3	Box 4	ATTAAT	1350	part of a conserved DNA module involved in light responsiveness
OsCBL3	Box 4	ATTAAT	1384	part of a conserved DNA module involved in light responsiveness
OsCBL3	Box 4	ATTAAT	1592	part of a conserved DNA module involved in light responsiveness
OsCBL3	TGACG-motif	TGACG	94	cis-acting regulatory element involved in the MeJA-responsiveness
OsCBL3	TGACG-motif	TGACG	431	cis-acting regulatory element involved in the MeJA-responsiveness
OsCBL3	ABRE	ACGTG	1531	cis-acting element involved in the abscisic acid responsiveness
OsCBL3	ABRE	GCCGCGTGGC	1873	cis-acting element involved in the abscisic acid responsiveness
OsCBL3	GT1-motif	GGTTAA	151	light responsive element

OsCBL3	LTR	CCGAAA	1849	cis-acting element involved in low-temperature responsiveness
OsCBL3	ACE	GCGACGTACC	427	cis-acting element involved in light responsiveness
OsCBL3	GARE-motif	TCTGTTG	1628	gibberellin-responsive element
OsCBL3	CCAAT-box	CAACGG	1636	MYBHv1 binding site
OsCBL3	G-Box	CACGTT	1531	cis-acting regulatory element involved in light responsiveness
OsCBL3	Sp1	GGGCGG	1926	light responsive element
OsCBL4	TCCC-motif	TCTCCCT	1928	part of a light responsive element
OsCBL4	GC-motif	CCCCCG	35	enhancer-like element involved in anoxic specific inducibility
OsCBL4	GC-motif	CCCCCG	417	enhancer-like element involved in anoxic specific inducibility
OsCBL4	Box 4	ATTAAT	1302	part of a conserved DNA module involved in light responsiveness
OsCBL4	TGACG-motif	TGACG	453	cis-acting regulatory element involved in the MeJA-responsiveness
OsCBL4	TGACG-motif	TGACG	668	cis-acting regulatory element involved in the MeJA-responsiveness
OsCBL4	TGACG-motif	TGACG	690	cis-acting regulatory element involved in the MeJA-responsiveness
OsCBL4	G-box	CACGAC	430	cis-acting regulatory element involved in light responsiveness
OsCBL4	G-box	GCCACGTGGA	996	cis-acting regulatory element involved in light responsiveness
OsCBL4	G-box	CACGTG	998	cis-acting regulatory element involved in light responsiveness
OsCBL4	G-box	CACGTG	1082	cis-acting regulatory element involved in light responsiveness
OsCBL4	G-box	TACGTG	1470	cis-acting regulatory element involved in light responsiveness
OsCBL4	G-box	TACGTG	1887	cis-acting regulatory element involved in light responsiveness
OsCBL4	CGTCA-motif	CGTCA	453	cis-acting regulatory element involved in the MeJA-responsiveness

OsCBL4	CGTCA-motif	CGTCA	668	cis-acting regulatory element involved in the MeJA-responsiveness
OsCBL4	CGTCA-motif	CGTCA	690	cis-acting regulatory element involved in the MeJA-responsiveness
OsCBL4	Box II	CCACG TGGC	996	part of a light responsive element
OsCBL4	ABRE	ACGTG	439	cis-acting element involved in the abscisic acid responsiveness
OsCBL4	ABRE	ACGTG	521	cis-acting element involved in the abscisic acid responsiveness
OsCBL4	ABRE	ACGTG	602	cis-acting element involved in the abscisic acid responsiveness
OsCBL4	ABRE	CACGT G	998	cis-acting element involved in the abscisic acid responsiveness
OsCBL4	ABRE	ACGTG	999	cis-acting element involved in the abscisic acid responsiveness
OsCBL4	ABRE	CACGT G	1082	cis-acting element involved in the abscisic acid responsiveness
OsCBL4	ABRE	ACGTG	1083	cis-acting element involved in the abscisic acid responsiveness
OsCBL4	ABRE	ACGTG	1471	cis-acting element involved in the abscisic acid responsiveness
OsCBL4	ABRE	ACGTG	1888	cis-acting element involved in the abscisic acid responsiveness
OsCBL4	A-box	CCGTCC	345	cis-acting regulatory element
OsCBL4	A-box	CCGTCC	388	cis-acting regulatory element
OsCBL4	A-box	CCGTCC	673	cis-acting regulatory element
OsCBL4	TCA-element	CCATCT TTTT	1632	cis-acting element involved in salicylic acid responsiveness
OsCBL4	CCAAT-box	CAACG G	715	MYBHv1 binding site
OsCBL4	G-Box	CACGTT	438	cis-acting regulatory element involved in light responsiveness
OsCBL4	G-Box	CACGTT	520	cis-acting regulatory element involved in light responsiveness
OsCBL4	G-Box	CACGTT	601	cis-acting regulatory element involved in light responsiveness
OsCBL4	G-Box	CACGT G	998	cis-acting regulatory element involved in light responsiveness
OsCBL4	G-Box	CACGT G	1082	cis-acting regulatory element involved in light responsiveness

OsCBL4	Sp1	GGGCGG	951	light responsive element
OsCBL4	LTR	CCGAAA	1814	cis-acting element involved in low-temperature responsiveness
OsCBL1	I-box	gGATAAGGTG	724	part of a light responsive element
OsCBL1	TGA-element	AACGAC	1016	auxin-responsive element
OsCBL1	P-box	CCTTTTG	223	gibberellin-responsive element
OsCBL1	GC-motif	CCCCCG	1663	enhancer-like element involved in anoxic specific inducibility
OsCBL1	GC-motif	CCCCCG	1785	enhancer-like element involved in anoxic specific inducibility
OsCBL1	GC-motif	CCCCCG	1857	enhancer-like element involved in anoxic specific inducibility
OsCBL1	TCT-motif	TCTTAC	1265	part of a light responsive element
OsCBL1	TGACG-motif	TGACG	126	cis-acting regulatory element involved in the MeJA-responsiveness
OsCBL1	TGACG-motif	TGACG	289	cis-acting regulatory element involved in the MeJA-responsiveness
OsCBL1	TGACG-motif	TGACG	555	cis-acting regulatory element involved in the MeJA-responsiveness
OsCBL1	TGACG-motif	TGACG	680	cis-acting regulatory element involved in the MeJA-responsiveness
OsCBL1	Box 4	ATTAAT	916	part of a conserved DNA module involved in light responsiveness
OsCBL1	Box 4	ATTAAT	924	part of a conserved DNA module involved in light responsiveness
OsCBL1	motif I	gGTACGTGGCG	368	cis-acting regulatory element root specific
OsCBL1	TCCC-motif	TCTCCCT	39	part of a light responsive element
OsCBL1	TCA-element	TCAGAAGAGG	818	cis-acting element involved in salicylic acid responsiveness
OsCBL1	TCA-element	CCATCTTTTT	1028	cis-acting element involved in salicylic acid responsiveness
OsCBL1	GT1-motif	GGTTAA	963	light responsive element

OsCBL1	ABRE	GCAACGTGTC	633	cis-acting element involved in the abscisic acid responsiveness
OsCBL1	ABRE	CACGTG	635	cis-acting element involved in the abscisic acid responsiveness
OsCBL1	ABRE	ACGTG	636	cis-acting element involved in the abscisic acid responsiveness
OsCBL1	ABRE	CACGTG	665	cis-acting element involved in the abscisic acid responsiveness
OsCBL1	ABRE	ACGTG	666	cis-acting element involved in the abscisic acid responsiveness
OsCBL1	ABRE	ACGTG	752	cis-acting element involved in the abscisic acid responsiveness
OsCBL1	ABRE	AACCCGG	771	cis-acting element involved in the abscisic acid responsiveness
OsCBL1	ABRE	GCCGCGTGGC	778	cis-acting element involved in the abscisic acid responsiveness
OsCBL1	ABRE	CACGTG	780	cis-acting element involved in the abscisic acid responsiveness
OsCBL1	ABRE	ACGTG	781	cis-acting element involved in the abscisic acid responsiveness
OsCBL1	ABRE	CACGTG	1624	cis-acting element involved in the abscisic acid responsiveness
OsCBL1	ABRE	ACGTG	1625	cis-acting element involved in the abscisic acid responsiveness
OsCBL1	Box II	CCACGTGGC	778	part of a light responsive element
OsCBL1	Box II	CCACGTGGC	779	part of a light responsive element
OsCBL1	CGTCA-motif	CGTCA	126	cis-acting regulatory element involved in the MeJA-responsiveness
OsCBL1	CGTCA-motif	CGTCA	289	cis-acting regulatory element involved in the MeJA-responsiveness
OsCBL1	CGTCA-motif	CGTCA	555	cis-acting regulatory element involved in the MeJA-responsiveness
OsCBL1	CGTCA-motif	CGTCA	680	cis-acting regulatory element involved in the MeJA-responsiveness
OsCBL1	G-box	ACACGTGGC	634	cis-acting regulatory element involved in light responsiveness
OsCBL1	G-box	CACGTG	635	cis-acting regulatory element involved in light responsiveness

OsCBL1	G-box	CACGTG	665	cis-acting regulatory element involved in light responsiveness
OsCBL1	G-box	CACGTC	751	cis-acting regulatory element involved in light responsiveness
OsCBL1	G-box	GCCACGTGGA	778	cis-acting regulatory element involved in light responsiveness
OsCBL1	G-box	CACGTG	780	cis-acting regulatory element involved in light responsiveness
OsCBL1	G-box	CACGAC	1155	cis-acting regulatory element involved in light responsiveness
OsCBL1	G-box	CACGTG	1624	cis-acting regulatory element involved in light responsiveness
OsCBL1	ATC-motif	AGTAATCT	1188	part of a conserved DNA module involved in light responsiveness
OsCBL1	G-Box	CACGTG	635	cis-acting regulatory element involved in light responsiveness
OsCBL1	G-Box	CACGTG	665	cis-acting regulatory element involved in light responsiveness
OsCBL1	G-Box	CACGTG	780	cis-acting regulatory element involved in light responsiveness
OsCBL1	G-Box	CACGTG	1624	cis-acting regulatory element involved in light responsiveness
OsCBL1	TC-rich repeats	GTTTTCTTAC	973	cis-acting element involved in defense and stress responsiveness
OsCBL1	Sp1	GGGCGG	700	light responsive element
OsCBL1	Sp1	GGGCGG	826	light responsive element
OsCBL1	Sp1	GGGCGG	1788	light responsive element
OsCBL1	Sp1	GGGCGG	1982	light responsive element
OsCBL1	ARE	AAACCA	77	cis-acting regulatory element essential for the anaerobic induction
OsCBL1	ARE	AAACCA	491	cis-acting regulatory element essential for the anaerobic induction
OsCBL1	ARE	AAACCA	895	cis-acting regulatory element essential for the anaerobic induction

OsCBL1	ARE	AAACCA	971	cis-acting regulatory element essential for the anaerobic induction
OsCBL1	ARE	AAACCA	1051	cis-acting regulatory element essential for the anaerobic induction
OsCBL1	ARE	AAACCA	1220	cis-acting regulatory element essential for the anaerobic induction
OsCBL1	ARE	AAACCA	1395	cis-acting regulatory element essential for the anaerobic induction
OsCBL1	CCAAT-box	CAACGG	1680	MYBHv1 binding site
OsCBL6	ARE	AAACCA	321	cis-acting regulatory element essential for the anaerobic induction
OsCBL6	Sp1	GGGCGG	1551	light responsive element
OsCBL6	Sp1	GGGCGG	1686	light responsive element
OsCBL6	Sp1	GGGCGG	1887	light responsive element
OsCBL6	ACE	GACACGTATG	1091	cis-acting element involved in light responsiveness
OsCBL6	AT-rich sequence	TAAAATACT	549	element for maximal elicitor-mediated activation (2copies)
OsCBL6	AT-rich sequence	TAAAATACT	606	element for maximal elicitor-mediated activation (2copies)
OsCBL6	LTR	CCGAAA	1768	cis-acting element involved in low-temperature responsiveness
OsCBL6	LTR	CCGAAA	1814	cis-acting element involved in low-temperature responsiveness
OsCBL6	AT-rich element	ATAGAAATCAA	135	binding site of AT-rich DNA binding protein (ATBP-1)
OsCBL6	AT-rich element	ATAGAAATCAA	231	binding site of AT-rich DNA binding protein (ATBP-1)
OsCBL6	TATC-box	TATCCCA	1801	cis-acting element involved in gibberellin-responsiveness
OsCBL6	G-box	CACGTC	936	cis-acting regulatory element involved in light responsiveness

OsCBL6	G-box	CACGAC	1462	cis-acting regulatory element involved in light responsiveness
OsCBL6	G-box	CACGTC	1822	cis-acting regulatory element involved in light responsiveness
OsCBL6	CGTCA-motif	CGTCA	99	cis-acting regulatory element involved in the MeJA-responsiveness
OsCBL6	CGTCA-motif	CGTCA	247	cis-acting regulatory element involved in the MeJA-responsiveness
OsCBL6	A-box	CCGTCC	1547	cis-acting regulatory element
OsCBL6	O2-site	GATGA(C/T)(A/G)TG(A/G)	581	cis-acting regulatory element involved in zein metabolism regulation
OsCBL6	ABRE	ACGTG	936	cis-acting element involved in the abscisic acid responsiveness
OsCBL6	ABRE	ACGTG	1822	cis-acting element involved in the abscisic acid responsiveness
OsCBL6	CAT-box	GCCACT	1019	cis-acting regulatory element related to meristem expression
OsCBL6	Box 4	ATTAAT	360	part of a conserved DNA module involved in light responsiveness
OsCBL6	Box 4	ATTAAT	394	part of a conserved DNA module involved in light responsiveness
OsCBL6	TGACG-motif	TGACG	99	cis-acting regulatory element involved in the MeJA-responsiveness
OsCBL6	TGACG-motif	TGACG	247	cis-acting regulatory element involved in the MeJA-responsiveness
OsCBL6	TCT-motif	TCTTAC	695	part of a light responsive element
OsCBL6	TCT-motif	TCTTAC	1049	part of a light responsive element
OsCBL6	GC-motif	CCCCCG	1414	enhancer-like element involved in anoxic specific inducibility
OsCBL6	GC-motif	CCCCCG	1581	enhancer-like element involved in anoxic specific inducibility
OsCBL6	GC-motif	CCCCCG	1603	enhancer-like element involved in anoxic specific inducibility
OsCBL6	GC-motif	CCCCCG	1631	enhancer-like element involved in anoxic specific inducibility
OsCBL6	P-box	CCTTTTG	56	gibberellin-responsive element

OsCBL6	TGA-element	AACGAC	1425	auxin-responsive element
OsCBL2	LTR	CCGAAA	482	cis-acting element involved in low-temperature responsiveness
OsCBL2	ATC-motif	TGCTATCCA	434	part of a conserved DNA module involved in light responsiveness
OsCBL2	G-Box	CACGTG	142	cis-acting regulatory element involved in light responsiveness
OsCBL2	TC-rich repeats	GTTTTCTTAC	1073	cis-acting element involved in defense and stress responsiveness
OsCBL2	Sp1	GGGCGG	1404	light responsive element
OsCBL2	Sp1	GGGCGG	1462	light responsive element
OsCBL2	Sp1	GGGCGG	1874	light responsive element
OsCBL2	Sp1	GGGCGG	1962	light responsive element
OsCBL2	CCAAT-box	CAACGG	1109	MYBHv1 binding site
OsCBL2	CCAAT-box	CAACGG	1385	MYBHv1 binding site
OsCBL2	ARE	AAACCA	634	cis-acting regulatory element essential for the anaerobic induction
OsCBL2	ARE	AAACCA	1496	cis-acting regulatory element essential for the anaerobic induction
OsCBL2	ABRE	CACGTG	142	cis-acting element involved in the abscisic acid responsiveness
OsCBL2	ABRE	ACGTG	143	cis-acting element involved in the abscisic acid responsiveness
OsCBL2	ABRE	AACCCGG	1654	cis-acting element involved in the abscisic acid responsiveness
OsCBL2	GT1-motif	GGTTAA	550	light responsive element
OsCBL2	GT1-motif	GGTTAA	552	light responsive element
OsCBL2	GT1-motif	GGTTAA	1035	light responsive element
OsCBL2	GT1-motif	GGTTAA	1657	light responsive element

OsCBL2	GT1-motif	GGTTAA	1659	light responsive element
OsCBL2	A-box	CCGTCC	1251	cis-acting regulatory element
OsCBL2	A-box	CCGTCC	1255	cis-acting regulatory element
OsCBL2	CGTCA-motif	CGTCA	1621	cis-acting regulatory element involved in the MeJA-responsiveness
OsCBL2	G-box	TAACACGTAG	139	cis-acting regulatory element involved in light responsiveness
OsCBL2	G-box	CACGTG	142	cis-acting regulatory element involved in light responsiveness
OsCBL2	TCCC-motif	TCTCCCT	1375	part of a light responsive element
OsCBL2	GC-motif	CCCCCG	1566	enhancer-like element involved in anoxic specific inducibility
OsCBL2	GC-motif	CCCCCG	1965	enhancer-like element involved in anoxic specific inducibility
OsCBL2	TCT-motif	TCTTAC	1073	part of a light responsive element
OsCBL2	Box 4	ATTAAT	456	part of a conserved DNA module involved in light responsiveness
OsCBL2	TGACG-motif	TGACG	1621	cis-acting regulatory element involved in the MeJA-responsiveness
OsCBL2	I-box	GTATAAGGCC	207	part of a light responsive element
OsCBL2	GCN4_motif	TGAGTCA	68	cis-regulatory element involved in endosperm expression
